# Diffuse Large B-cell Lymphoma Mimicking Pancreatic Carcinoma and the Use of Immunohistochemistry in Resolving the Diagnostic Dilemma After Postmortem Examination: A Case Report

**DOI:** 10.7759/cureus.44227

**Published:** 2023-08-27

**Authors:** Ifeanyichukwu D Nwanji, Babatope L Awosusi, Alexander O Odigwe, Mustapha A Ajani

**Affiliations:** 1 Pathology, University College Hospital, Ibadan, NGA; 2 Pathology and Laboratory Medicine, King Khaled Majma'ah Hospital, Al Majma'ah, SAU; 3 Pathology, Lily Hospitals, Warri, NGA; 4 Pathology, University College Hospital, University of Ibadan, Ibadan, NGA

**Keywords:** postmortem examination, non-hodgkin lymphoma (nhl), pancreatic carcinoma, disseminated lymphoma, large b-cell lymphoma

## Abstract

Diffuse large B-cell lymphoma is the most common lymphoma, accounting for 30% of all non-Hodgkin lymphomas; they can grow rapidly and often present as masses infiltrating tissues or obstructing organs.

We report the case of a 58-year-old female who presented with a one-month history of generalized body weakness and weight loss with a two-week history of yellowness of the eyes, fever, dyspnea, and bilateral leg swelling. Examination at presentation revealed pallor, fever, jaundice, hepatomegaly, and bilateral pitting pedal edema. Laboratory investigations revealed severe anemia, deranged clotting profile, azotemia, elevated liver enzymes, and elevated tumor markers CA125, CEA, and CA 19.9. Abdominal ultrasound showed hepatomegaly and a large head of the pancreas. The initial diagnosis was obstructive jaundice due to carcinoma of the head of the pancreas. Despite all care offered, her clinical condition deteriorated until she died on the 12th day of admission. A postmortem examination showed a mass in the head of the pancreas and bile duct, enlarged para-aortic and mesenteric lymph nodes with ascites, pericardial nodules, and bronchopneumonia. Histological and immunohistochemical analysis of postmortem biopsies confirmed the diagnosis of a diffuse large B cell lymphoma.

This case highlights the diagnostic dilemma often seen in disseminated diffuse large B-cell lymphoma. The patient presented with features referable to different organs and systems. If detected early, most cases respond to standard immuno-chemotherapy. However, it can also become rapidly fatal and ultimately lead to death, as seen in this case.

## Introduction

Diffuse large B-cell lymphoma is the most common and accounts for approximately 30% of non-Hodgkin lymphoma cases [1]. Extranodal lymphoma occurs in about 40% of patients and has been described in almost every organ and tissue in the body [2]. In decreasing order of occurrence, the spleen, liver, gastrointestinal tract, pancreas, abdominal wall, genitourinary tract, adrenal gland, peritoneal cavity, and biliary tract are affected [2]. The involvement of the bile duct in lymphoma cases is rare and usually a secondary manifestation of systemic lymphoma [2]. Non-Hodgkin's lymphoma (NHL) accounts for 1% to 2% of all cases of malignant biliary obstruction, while the incidence of obstructive jaundice as an initial presentation of NHL is also only seen in 1% to 2% of all patients [3-4]. Nguyen described the first case of non-Hodgkin lymphoma arising from the bile duct in 1982 [5].

Cardiac metastases occur in 20% to 25% of patients with lymphoma [6]. Some authors have described primary cardiac lymphomas with pericardial effusion, arrhythmias, and heart failure [6].

Here, we present the case of a 31-year-old Nigerian female who developed obstructive jaundice and congestive cardiac failure. The patient's condition deteriorated rapidly with eventual demise. Diffuse large B-cell lymphoma was diagnosed with postmortem examination, tissue biopsy, and immunohistochemistry.

## Case presentation

Clinical history and examination findings

The decedent was a middle-aged woman who presented to the Emergency Department of the University College Hospital, Ibadan, with a one-month history of generalized body weakness, a two-week history of yellowness of the eyes, and five days of fever. There was breathlessness on moderate exertion but no orthopnea or paroxysmal nocturnal dyspnea. Yellowish discoloration of the eyes progressively deepened with associated pruritus. The fever was high-grade and intermittent and relieved by taking analgesics. There were additional symptoms of significant weight loss and bilateral leg swelling.

She was a trader and was not known to have any chronic medical illnesses. There was no family history of abdominal, pelvic, or blood malignancies. She had no known pre-existing medical conditions or potential risk factors for malignancy. 

On examination at presentation, she was conscious but acutely ill-looking, moderately pale, febrile (with a temperature of 38.5°C), mildly dehydrated, and had bilateral pedal edema. Cardiovascular system examination showed tachycardia (with a pulse rate of 110 beats per minute) and hypotension (with a blood pressure of 100/60 mmHg).

Chest examination showed tachycardia (with a respiratory rate of 28 cycles per minute). The liver was palpably enlarged 8 cm below the coastal margin.

Investigation findings

She was severely anemic, with a pack cell volume of 18% and a microcytic hypochromic blood film. She had a deranged clotting profile with a prothrombin time of 25.9 sec. She had azotemia with elevated urea and creatinine. Liver function was also deranged with severe conjugated hyperbilirubinemia, elevated alkaline phosphatase, and Gamma-glutamyl transferase. CA 125, CEA, and CA 19.9 tumor marker levels were all elevated. Bone marrow examination revealed erythroid hyperplasia. However, there were no blasts or plasma cell dyscrasia.

Abdominopelvic ultrasonography showed an enlarged liver with a craniocaudal span of 19.6 cm, a smooth outline with homogenous parenchymal echoes, no intrahepatic mass or biliary ductal dilatation; the spleen was enlarged (with a span of 12.4 cm) with mild ascites as well as markedly distended gallbladder with sludge. Kidneys were normal-sized. The pancreatic head appeared enlarged with a lobulated outline measuring 6.4x4.5cm in dimension. 

Treatment and outcome

The presumptive diagnosis was obstructive jaundice secondary to carcinoma of the head of the pancreas. She received three units of packed red blood cells. She also received two units of fresh frozen plasma, intravenous vitamin K, and empirical antimicrobials. She was worked up for exploratory laparotomy with possible pancreatic bypass surgery. 

While on admission, the fever persisted with an intermittent pattern despite antibiotics. Pedal edema worsened while renal function declined. In the second week of inpatient care, she had an episode of tonic-clonic convulsions involving the right side of the face and the right upper extremities. She became unconscious and was moved into the intensive care unit, where she was intubated and mechanically ventilated.

On the twelfth day of hospital admission, her clinical condition deteriorated. She had repeated cardiac arrests and was certified dead after all efforts to resuscitate her proved abortive. Consent for autopsy was obtained and assented to by relatives.

Autopsy presentation

At autopsy, we received the body of a middle-aged woman who was moderately pale and had bilateral pitting pedal edema up to the knee. There was no palpable peripheral lymphadenopathy. The abdomen is palpably enlarged, with 2 L of serous ascites noted. Both lungs were heavy and weighed 912 g on the right and 1162 g on the left. There was evidence of pneumonic consolidation in both lungs.

**Figure 1:**
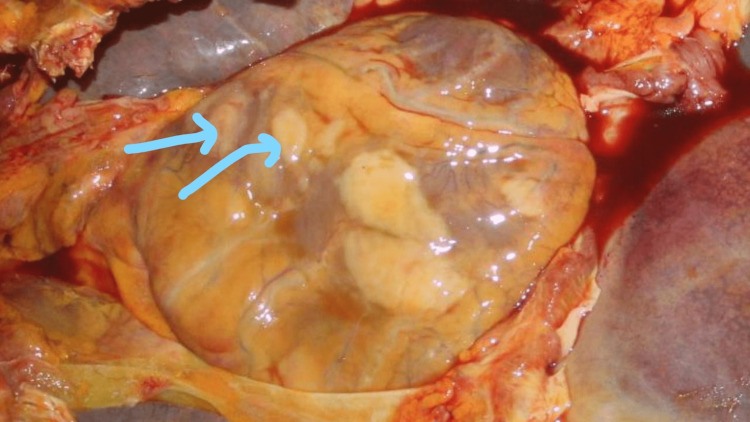
Photomicrograph showing the heart in-situ The blue arrows show greyish-white tumor nodules on the pericardial surface of the heart

The heart weighed 315 g with nodular, slightly elevated pale-yellow masses on the pericardial surface involving the right atrium and ventricle ranging in size from 2x1.5cm to 4x3 cm (Figure 1). The cut sections through the heart show similar lesions within the myocardium.

**Figure 2:**
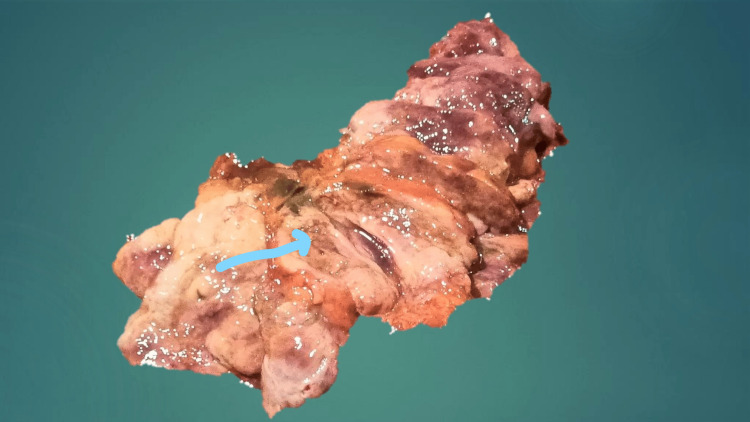
Photomicrograph showing an enlarged pancreas The blue arrow shows pale grey cut surfaces of the pancreatic mass

The liver was enlarged and weighed 2087 g. Cut sections showed a nutmeg appearance. The gallbladder lumen contained bile and was free of gallstones. The anterior wall of the gallbladder showed few nodular lesions ranging in dimension from 1x0.5 cm to 2x1.5 cm. The biliary tree was not patent due to a pancreatic mass involving the entire length, weighing 800 g (60-135 g) and measuring 20x8x8xm. This is shown below in Figure 2. 

There were about 14 enlarged mesenteric nodes with varying sizes from 0.5 cm to 4 cm in their widest diameter. Also noted were multiple para-aortic nodes, the largest of which measures 6x4x4cm. Cut sections through these lymph nodes show a fish-flesh appearance.

Histology and immunohistochemistry

Sections of the para-aortic and mesenteric lymph nodes showed near-total effacement of nodal architecture by markedly pleomorphic cells disposed mainly in sheets. The cells have intermediate to large-sized hyperchromatic nuclei, clumped chromatin, and scanty to moderate amphophilic cytoplasm. There were interspersed tumor giant cells. There was extensive infiltration of the pancreas, myocardial tissue, uterine myometrium, and gallbladder by similar tumor cells. Histological sections of the lung showed features consistent with severe bronchopneumonia. Sections of the liver showed features of chronic passive congestion. 

**Figure 3:**
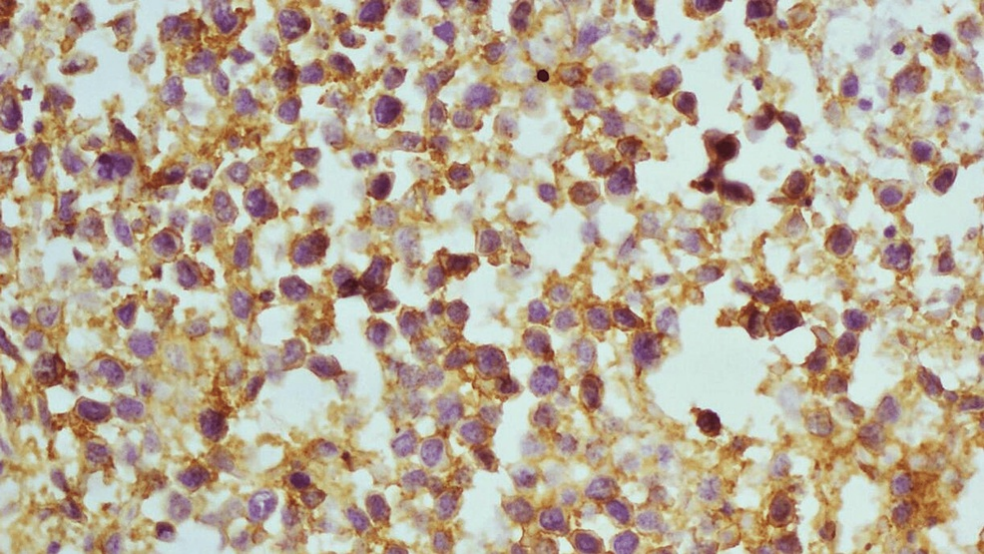
Photomicrograph shows tumor cells positive for CD 45 antibody (as indicated by the golden brown color)

**Figure 4:**
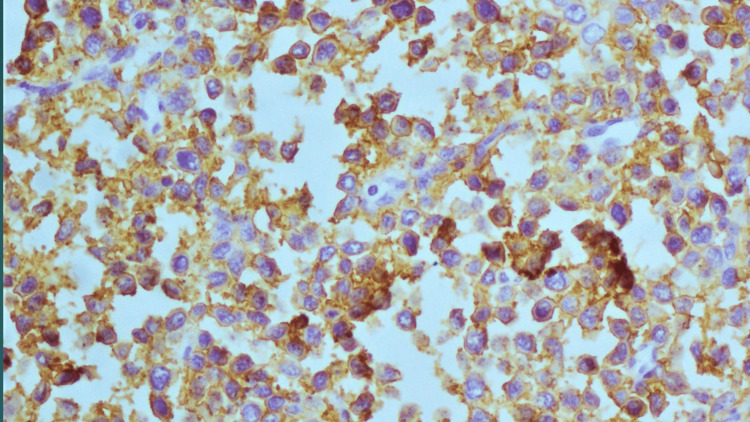
Photomicrograph shows tumor cells positive for CD20 antibody (as indicated by the golden brown color)

The differential diagnosis after examination of tissue at the light microscopic level includes carcinoma, lymphoma, and rhabdomyosarcoma. Tissue biopsies taken from the pancreas were then subjected to immunohistochemistry using a panel of antibodies CD45, CD20, and AE1/AE3, Epithelial membrane antigen, desmin, and myogenin. The tumor cells were positive for CD45 and CD20 (Figures 3, 4). Positivity for CD45 confirms the lymphoid origin of the tumor cells, while positivity for CD20 further confirms the lymphoid cells to be of B-cell origin. Epithelial membrane antigen was positive in residual pancreatic acini but not in the tumor cells. AE1 and AE3 were negative in the tumor cells; this excludes the possibility of carcinoma. Desmin and myogenin were negative; this excludes the possibility of a rhabdomyosarcoma. 

The final autopsy diagnosis was disseminated diffuse large B-cell lymphoma based on the histomorphologic findings and immunohistochemical staining characteristics of the tumor cells. 

## Discussion

This case highlights diagnostic dilemmas that arose clinically and at autopsy due to disseminated diffuse large B-cell lymphoma. The patient presented with features referable to many organs/systems. The initial onset of weakness and fatigue pointed to a blood dyscrasia. However, bone marrow findings showed only erythroid hyperplasia. The absence of peripheral lymphadenopathy is also worthy of note in this case. The development of jaundice and a pancreatic mass with severe derangement of liver function suggested pancreatic cancer. To further confound the diagnosis, there were elevated tumor markers.

There was also a similar diagnostic dilemma at autopsy. Multiple myocardial masses grossly resembling rhabdomyosarcoma were noted. In addition to para-aortic and mesenteric lymphadenopathy, there was a grossly enlarged pancreas and grossly visible tumor nodules on the gallbladder. Histologically, while some of the tumours were reminiscent of atypical lymphoid cells, there was an admixed population of cells with extreme cellular and nuclear pleomorphism. Only on immunohistochemistry was a conclusive diagnosis possible with findings of a CD45 and CD20 positivity, in keeping with a diffuse large B-cell lymphoma. Epithelial membrane antigen (EMA), myogenin, and desmin negativity ruled out carcinoma and rhabdomyosarcoma.

There have been some case reports of obstructive jaundice complicating non-Hodgkin lymphoma [7,8]. Fadiora et al. described a patient with primary ovarian lymphoma presenting with obstructive jaundice, among other features [9]. Cardiac involvement by NHL is uncommon, with a prevalence rate of 8.7% to 20% seen in autopsy series [10]. However, presentation with features of both obstructive jaundice and cardiac involvement is rare. This case highlights the challenges this can pose to diagnosis and treatment. If detected early, high-grade NHLs such as this index case show response to chemotherapeutic agents. Unfortunately, a definitive diagnosis was possible after postmortem examination due to late presentation and diagnostic dilemmas. The diagnostic challenges led to a delay in commencing appropriate treatment, which led to the eventual demise of the patient. 

## Conclusions

This case highlights the diagnostic dilemma often seen in disseminated diffuse large B cell lymphoma. The patient presented with features referable to different systems, leading to a delay in making a definitive diagnosis with subsequent deterioration in clinical condition and eventual demise. If detected early, most cases of diffuse large B cell lymphoma respond to standard immunotherapy and chemotherapy. However, it can be rapidly fatal if there is a delay in diagnosis, as presented in this index case report.

## References

[1] Swerdlow SH, Campo E, Pileri SA (2016). The 2016 revision of the World Health Organization classification of lymphoid neoplasms. Blood.

[2] Lee WK, Lau EW, Duddalwar VA, Stanley AJ, Ho YY (2008). Abdominal manifestations of extranodal lymphoma: spectrum of imaging findings. AJR Am J Roentgenol.

[3] Chaudhari D, Khan S, Saleem A, Taylor T, Reddy C, Borthwick T, Young M (2013). Obstructive jaundice as an initial manifestation of non-hodgkin lymphoma: treatment dilemma and high mortality. Case Rep Med.

[4] Odemiş B, Parlak E, Başar O, Yüksel O, Sahin B (2007). Biliary tract obstruction secondary to malignant lymphoma: experience at a referral center. Dig Dis Sci.

[5] Lokich JJ, Kane RA, Harrison DA, McDermott WV (1987). Biliary tract obstruction secondary to cancer: management guidelines and selected literature review. J Clin Oncol.

[6] Amirimoghaddam Z, Khoddami M, Nayeri ND, Molaee S (2010). Hodgkin's lymphoma presenting with heart failure: a case report. J Med Case Rep.

[7] Hashimoto M, Umekita N, Noda K (2008). Non-Hodgkin lymphoma as a cause of obstructive jaundice with simultaneous extrahepatic portal vein obstruction: a case report. World J Gastroenterol.

[8] Zakaria A, Al-Obeidi S, Daradkeh S (2017). Primary non-Hodgkin's lymphoma of the common bile duct: a case report and literature review. Asian J Surg.

[9] Fadiora SO, Mabayoje VO, Oboro VO, Ojemakinde KA, Bello TO, Adeniji AA (2008). Ovarian non-Hodgkin's lymphoma presenting as obstructive jaundice - a case report. Niger Postgrad Med J.

[10] O'Mahony D, Peikarz RL, Bandettini WP, Arai AE, Wilson WH, Bates SE (2008). Cardiac involvement with lymphoma: a review of the literature. Clin Lymphoma Myeloma.

